# The ECM-Cell Interaction of Cartilage Extracellular Matrix on Chondrocytes

**DOI:** 10.1155/2014/648459

**Published:** 2014-05-18

**Authors:** Yue Gao, Shuyun Liu, Jingxiang Huang, Weimin Guo, Jifeng Chen, Li Zhang, Bin Zhao, Jiang Peng, Aiyuan Wang, Yu Wang, Wenjing Xu, Shibi Lu, Mei Yuan, Quanyi Guo

**Affiliations:** Institute of Orthopedics, Chinese PLA General Hospital, Beijing 100853, China

## Abstract

Cartilage extracellular matrix (ECM) is composed primarily of the network type II collagen (COLII) and an interlocking mesh of fibrous proteins and proteoglycans (PGs), hyaluronic acid (HA), and chondroitin sulfate (CS). Articular cartilage ECM plays a crucial role in regulating chondrocyte metabolism and functions, such as organized cytoskeleton through integrin-mediated signaling via cell-matrix interaction. Cell signaling through integrins regulates several chondrocyte functions, including differentiation, metabolism, matrix remodeling, responses to mechanical stimulation, and cell survival. The major signaling pathways that regulate chondrogenesis have been identified as wnt signal, nitric oxide (NO) signal, protein kinase C (PKC), and retinoic acid (RA) signal. Integrins are a large family of molecules that are central regulators in multicellular biology. They orchestrate cell-cell and cell-matrix adhesive interactions from embryonic development to mature tissue function. In this review, we emphasize the signaling molecule effect and the biomechanics effect of cartilage ECM on chondrogenesis.

## 1. What Is ECM?

In biology, the extracellular matrix (ECM) is the extracellular part of multicellular structure (e.g., organisms, tissues, and biofilms) that typically provides structural and biochemical support to the surrounding cells [1]. Because multicellularity evolved independently in different multicellular lineages, the composition of ECM varies between multicellular structures; however, cell adhesion, cell-to-cell communication, and differentiation are common functions of the ECM [2].

Cartilage ECM is composed primarily of COLII and large networks of PGs that contain GAG such as HA and CS. Because cartilage shows little tendency for self-repair, injuries remain unhealed for years and can lead to further degeneration [3]. Cartilaginous ECM is remodeled continuously by a combination of production, degradation by matrix metalloproteinases (MMPs), and inhibition of MMPs activity by tissue inhibitors of MMPs [4]. The ECM of articular cartilage is a unique environment. ECM components through their action on integrin clustering are involved in cell adhesion, cortical actin cytoskeleton organization, and cell spreading [5].

**Figure 1:**
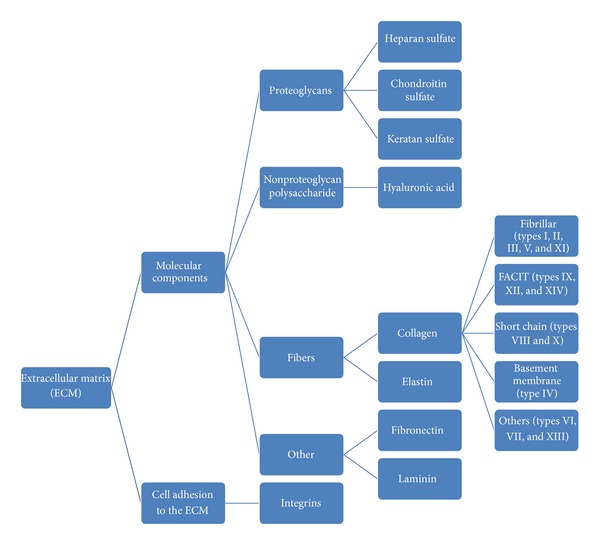


The ECM is composed of large proteoglycans (PGs) that contain glycosaminoglycan (GAG), hyaluronic acid (HA), fibers, and other molecular components about fibronectin and laminin. Fibers contain elastin and collagen that include fibrillar (types I, II, III, V, and XI), FACIT (types IX, XII, and XIV), short chain (types VIII and X), basement membrane (type IV), and others (types VI, VII, and XIII) [6] (Figure 1). In the ECM, especially the basement membrane, the multidomain proteins perlecan, agrin, and COLXVIII are the main proteins to which heparan sulfate attaches [7]. At last, there are important molecular components called integrins. Integrins are transmembrane receptors that mediate the attachment between a cell and its surroundings, such as other cells or the extracellular matrix (ECM). In signal transduction, integrins pass information about the chemical composition and mechanical status of the ECM into the cell. Therefore, in addition to transmitting mechanical forces across otherwise vulnerable membranes, they are involved in cell signaling and the regulation of cell cycle, shape, and motility.

## 2. What Is the Function of ECM?

Because of its diverse nature and composition, the ECM can have many functions, such as providing support, segregating tissues, and regulating intercellular communication. The ECM regulates a cell's dynamic behavior. In addition, it stores a wide range of cellular growth factors and acts as a local depot for them. Changes in physiological conditions can trigger protease activities that cause the local release of such depots. This situation allows for the rapid and local growth-factor-mediated activation of cellular functions. The formation of the ECM is essential for processes such as growth, wound healing, and fibrosis. PGs have a net negative charge that attracts positively charged sodium ions which attracts water molecules via osmosis. PGs can keep the ECM and resident cells hydrated. PGs may also help trap and store growth factors within the ECM. Once secreted, the molecules aggregate with the existing matrix. Resident cells intracellularly produce the components of the ECM via exocytosis [6].

Articular cartilage ECM plays a crucial role in regulating chondrocyte functions via cell-matrix interaction, organized cytoskeleton, and integrin-mediated signaling. The ECM has a significant effect on the swelling behavior and osmotic environment of chondrocytes [8]. Factors produced by chondrocytes can affect the synthesis of the ECM. These factors are ILs, basic fibroblast growth factor (BFGF), bone morphogenic proteins (BMPs), and insulin-like growth factor (IGF). Cell signaling mediated by integrin regulates several chondrocyte functions, including differentiation, matrix remodeling, responses to mechanical stimulation, and cell survival [9–11]. CS and HA influence the proliferation and differentiation of chondrocytes. Scaffolds composed of COLII, CS, and HA may create an environment that can preserve the normal phenotype of cells to promote regeneration of cartilage-like constructs [12]. CS contributes to the tensile strength of cartilage, tendons, ligaments, and walls of the aorta. Low-molecular-weight isoforms of the aggrecanases are responsible for the cytokine-induced proteolysis of aggrecan in a porcine chondrocyte culture system [13, 14]. Abnormal contact between chondrocytes and the ECM has serious consequences. Chondrocytes isolated from Rac1-deficient growth plates show reduced adhesion to COLII and fibronectin [15]. To provide a larger space to allow for cell proliferation and generation of new ECM, we found that a COLII scaffold composed of collagen with genipin is similar to natural ECM; the application of CS can increase mRNA and DNA biosynthesis and promote cell metabolism the same as with the acid mucopolysaccharide HA, with strong bonding and hydrophilic properties, to retain moisture, so that it better resembles the natural ECM and promotes cell proliferation [12].

Interactions between chondrocytes and the ECM regulate many biological processes important to homeostasis and repair of articular cartilage, including cell attachment, growth, differentiation, and survival. Integrins have two main functions: (1) attachment of the cell to the ECM and (2) signal transduction from the ECM to the cell.

However, they are also involved in a wide range of other biological activities, including immune patrolling, cell migration, and binding to cells by certain viruses, such as adenovirus, echovirus, hantavirus, and foot and mouth disease viruses.

A prominent function of the integrins is seen in the molecule GPIIbIIIa, an integrin on the surface of blood platelets (thrombocytes) responsible for attachment to fibrin within a developing blood clot. This molecule dramatically increases its binding affinity for fibrin/fibrinogen through association of platelets with exposed collagens in the wound site. Upon association of platelets with collagen, GPIIbIIIa changes shape, allowing it to bind to fibrin and other blood components to form the clot matrix and stop blood loss. Integrins are adhesion receptor heterodimers that transmit information from the ECM to the cell through activation of cell signaling pathways. The integrins are a large family of heterodimeric cell adhesion receptors involved in cell-cell and cell-matrix interactions [16, 17].

## 3. ECM and Cell Interaction

The general concept of chondrogenesis is as follows. In embryogenesis, the skeletal system is derived from the mesoderm germ layer. Chondrogenesis is the process by which cartilage is formed from condensed mesenchyme tissue, which differentiates into chondrocytes and begins secreting the molecules that form the ECM.

The relationship between ECM and chondrogenesis should be discussed here. The 3D environment of the ECM guides the morphogenesis of tissue types with anisotropic structures [18]. Chondrocyte differentiation is a multistep process characterized by successive changes in cell morphologic features and gene expression. Early in fetal development, the greater part of the skeleton is cartilaginous. This temporary cartilage is gradually replaced by bone (endochondral ossification), a process that ends at puberty. In contrast, the cartilage in the joints remains unossified during the whole life and is, therefore, permanent. During the early phase of the chondrocyte life cycle, cell-cell adhesion occurs via molecules such as N-cadherin. At later stages, such as in growth-plate chondrocytes, adhesion signaling occurs from ECM proteins via integrin and other ECM receptors. Cell-matrix interactions are also important for chondrogenesis.

### 3.1. Signals and Integrins

The major signaling pathways that regulate chondrogenesis must play an important role through the cell-matrix interaction. These molecules are bound to plasma membrane or intracellular receptors and are interpreted by complex molecular pathways that use specific combinations of a cell or tissue-specific signaling toolkit, and, by eventually converging on transcription factors, they induce changes in gene expression. These signals are required to adjust the cellular metabolism to the needs of the tissue and/or organism or to affect the fate of cells: proliferation, differentiation, or apotosis [19] through wnt signal, nitric oxide signal, retinoic acid (RA) signal, and protein kinase C (PKC).

*Wnt9a* was shown to be required for chondrocyte proliferation and mediolateral intercalation, cellular mechanisms that mediate extension during zebrafish palate morphogenesis [20].* Frzb* and* fzd7a* are dispensable for directed migration of the bilateral trabeculae, but necessary for the convergence and extension of the palatal elements, where the extension process is mediated by chondrocyte proliferation, morphologic change, and intercalation.* Bapx1* was specifically downregulated in the* wnt9a/frzb/fzd7a* morphants. Overexpression of* bapx1* can partially rescue the lower jaw elements in* wnt9a*,* frzb*, and* fzd7a* morphants [21].

Nitric oxide (NO) was recognized as an important second messenger signaling molecule generated from metabolism of L-arginine by the nitric oxide synthase (NOS) family of enzymes [22]. Nitric oxide synthase inhibitor 1-(2-[trifluoromethyl] phenyl) imidazole (TRIM) can disrupt chondrogenic differentiation. So TRIM-treated embryo only formed scattered chondrocyte clusters. TRIM treatment could be reasoned by several developmental events, such as failure in identity specification within changes in cell proliferation and survival, and/or defects in chondrogenic differentiation. NO might function upstream of histone acetylation and/or through nonacetylation pathways (e.g., through S-nitrosylation; or NO may directly target the expression of chondrogenic genes). TRIM inhibited chondrogenic differentiation, which were mediated through impaired nitric oxide (NO) production without appreciable effect on global protein S-nitrosylation. TRIM perturbed Hox gene patterning and caused histone hypoacetylation [23]. NO regulates cartilage degradation by causing dedifferentiation and apoptosis of chondrocytes via activation of ERK1/2 and p38 [24].

RA is responsible for most of the activity of vitamin A and saves visual pigment effects that require retinal (retinaldehyde) and cell metabolism effects that may require retinol itself. Also, some biochemical functions necessary for fertility in vitamin A deficient male and female mammals originally appeared to require retinol for rescue, but this is due to a requirement for local conversion of retinol to RA, as administered RA does not reach some critical tissues unless given in high amounts. RA significantly increased the motility of neural crest cells, as shown by the wound-healing assay, and inhibited their proliferation. Cartilage elements originate from midbrain neural crest cells. RA can cause abnormal craniofacial cartilage development in other vertebrates, resulting in dose- and stage-dependent losses of* dlx* homeobox gene expression in several regions of the embryo [25, 26].

During chondrogenesis, reversible phosphorylation of key target proteins is of particular importance during this process. Among protein kinases known to be involved in these pathways, PKC subtypes play pivotal roles. PKC is a quintessential regulator of chondrogenesis. PKCs regulate the chondrocyte phenotype via the actin cytoskeleton. PKC exerts its chondrogenesis-promoting effect via the ERK-MAPK pathway. PKC mediates chondrogenesis via the ERK1/2 pathway. Chondrocyte de- and redifferentiation are regulated by PKC and MAPK signaling. PKC mediates the effects of IGF-1 and EGF during chondrogenesis. PKC-dependent regulation of chondrogenesis is via cell adhesion molecules [19].

In all, 24 unique integrin dimers are formed* in vivo* from the 18*α* and 8*β* subunits found in mammalian cells. The composition of the ECM is expressed in a given cell type. Integrins and cell signals can regulate cell shape and affinity. Chondrocytes express a subset of integrin subunits including fibronectin receptors, a laminin receptor, and collagen receptors [10, 27–32]. The *β*1 chain is a component of most chondrocyte integrins. Cartilage-specific deactivation of the *β*1-integrin gene results in severe changes in the cartilage phenotype [33]. Chondrocytes from knock-out mice show abnormal cell shape, reduced proliferation, and deregulated expression of cell-cycle proteins, including D-type cyclins and cyclin-dependent kinase inhibitors.* In vitro* experiments also suggest that the loss of *β*1 and *α*n*β*5 integrin promotes apoptosis in growth-plate chondrocytes and that antibodies against *β*1, *α*2, or *α*3 integrin [33–35] repress hypertrophic differentiation and decrease chondrocyte survival.

Integrin-mediated activation of members of the mitogen-activated protein kinase family plays a key role in transmitting signals regulating chondrocyte gene expression. Some research has verified with isotope-labeled monoclonal antibodies that chondrocyte phenotype remains may be due to the attachment mediated via integrin, including members of both the *β*1 and *β*3 subunit families. Then, chondrocytes showed significant attachment to fibronectin matrix Gla protein, osteopontin, bone sialoprotein II, vitronectin, and COLII and VI [36, 37], which suggests a link between matrix synthesis and integrin expression in chondrocytes. Chondrocytes express several members of the integrin family, including *α*5*β*1, the primary chondrocyte receptor for fibronectin. The *α*5*β*1 integrin provides matrix survival signals for normal and osteoarthritic human articular chondrocytes, to prevent apoptosis. Therefore, *β*-integrin-mediated chondrocyte-ECM interactions are decreased in osteoarthritic cartilage, which suggests that perturbations of chondrocyte-matrix signaling occurs during OA [10, 38, 39]. *β*1 integrin, the protein encoded by the ITGB1 gene (also known as CD29 and VLAB) [17], is a multifunctional protein involved in cell-matrix adhesion, cell signaling, cell adhesion, protein binding, and receptor-mediated activity. The *β*1-integrin family of cell-surface receptors appears to play a major role in mediating cell-matrix interactions that are important in regulating these fundamental processes.

Degradation of HA results in chondrocyte aggregation and then reduces chondrocyte apoptosis. As well, *β*1-integrin-collagen interaction reduces chondrocyte apoptosis [40], to achieve their goals by antagonizing hyaluronidase. So, like integrin-deficient chondrocytes, adhesion to the ECM decreased in Flnb (−/−) chondrocytes, and inhibition of *β*1 integrin in these cells further impaired cell spreading [41]. TGF-*β*1 and integrin stimuli interact before* Smad2* and -*3* phosphorylation in the cytoplasm of chondrocytes, which regulates the expression of ECM components in chondrocytes. Under culture and seeding conditions, *β*1, *α*5*β*1, and *α*v*β*5 integrins [42] mediate human chondrocyte adhesion to cartilage. These chondrocyte integrins have a potential role in the initial adhesion and retention of chondrocytes at a cartilage defect site. The fibronectin receptor (*α*5*β*1 integrin), in conjunction with its ligand fibronectin, the GPIIb/IIIa receptor and the integrin-linked kinase, integrin cytoplasmic-domain-associated protein 1, and CD47 pathway play a pivotal role in dedifferentiation of chondrocytes [16]. TGF-*β*3, MMP9, MMP13 [43–46], and vascular endothelial growth factor are key regulators for remodeling cartilage tissues. They coordinate matrix degradation and the recruitment and differentiation of osteoprogenitors. IL-1 receptor antagonist upregulates major components of the cartilage ECM genes, so we can use it to protect the ECM for anti-inflammatory and chondroprotective therapy.

### 3.2. Factors and Enzymes

After disruption of cell-matrix interactions and lack of growth factors, certain cells are selected and channelled through proliferation into the new stable phenotype. Chondrocyte mechanoreceptors may incorporate *β*1-integrins and mechanosensitive ion channels linked with key ECM, cytoskeletal, and signaling proteins to maintain the chondrocyte phenotype, prevent chondrocyte apoptosis, and regulate chondrocyte-specific gene expression [17, 47]. Tumor necrosis factor *α* (TNF-*α*) and interleukins-1*β* (IL-1*β*) cause the release of the stress-injury-related protein to relieve mechanical damage. TNF and IL-1 or anti-Fas antibody growth-regulated oncogene *α* in ECM can induce chondrocyte apoptosis. Chondrocyte apoptosis and caspase-3 activity are associated [9, 48]. IGF-1 is known to inhibit the catabolic effects of IL-1 on PG synthesis in cartilage explants and suppresses the degradation of ECM components by reducing matrix metalloproteinase-1 (MMP-1) and MMP-8 expression and activity [49].

Apoptosis is programmed cell death. Apoptotic cells take the initiative of cell death (necrosis). A disease such as OA can lead to apoptosis. OA results from the aberrant production of inflammatory mediators (cytokines and chemokines) and effectors (MMPs and reactive oxygen and nitrogen species) by chondrocytes [48]. Cartilage oligomeric matrix protein (COMP) plays an important role in cartilage cell-matrix interactions. COMPs induce the survival of the inhibitor of apoptosis family of proteins to lead to the strong inhibition of chondrocyte apoptosis by blocking the activation of caspase-3. The synthesis of COMP is regulated by transforming growth factor (TGF) in these 2 regions of the human articular cartilage [50–52]. COMP mutation has a great impact. Although it is a small molecule in the ECM, its mutation is the major reason for pseudoachondroplasia. COMP specifically locates in some cells of the rough endoplasmic reticulum and has toxic effects on chondrocyte precursors, thus hindering the formation of cartilage and bone [53–59]. A certain amount of strontium and COMP can maintain the structural integrity of the cartilage collagen and fibronectin [52, 60].

### 3.3. Biomechanics

In terms of biomechanics, cartilage tissue can remodel its ECM in response to alterations in functional demand. The pericellular matrix (PCM) is a narrow tissue region surrounding chondrocytes in articular cartilage, which together with the enclosed cell(s) has been termed the “chondron” [8]. The PCM is rich in fibronectin, PGs (e.g., aggrecan, HA, and decorin), and collagen (types II, VI, and IX) but, as compared with the ECM, is defined primarily by the presence of COLVI. The mechanical properties of PCM relative to those of the ECM can significantly affect the micromechanical environment of the chondrocyte. Changes in the properties of the PCM with osteoarthritis (OA) may alter the stress-strain and fluid-flow environment of chondrocytes [8, 61, 62].

OA leads to the degradation of the PCM and then alters the cellular environment of cartilage in terms of macroscopic loading features and material properties of the ECM and the chondron. At the microscale, estimates of Young's modulus of the PCM range from about 24 to 59 kPa by the axisymmetric boundary element method. Therefore, the PCM may have an important role in modulating the mechanical environment of the chondrocyte [63, 64]. Compressed damage by overloading the integrity of the cartilage ECM may cause cell membrane damage and eventually cell death. TNF and IL-1 cause the release of the stress-injury-related protein to relieve mechanical damage [65, 66].

## 4. Conclusions

The survey of the considerable domain of definition, components, and ECM-cell interaction of ECM can indicate the amount of knowledge accumulated and the directions of research and applications. It is evident that we are only at the beginning of understanding the precise role of cell-matrix interaction during chondrogenesis and how they are regulated. There are a lot of unexplored fields in this area; exploiting these novel approaches and applying them to not only healthy but also inflammatory chondrocytes may enable us to halt or even reverse disease progression. Shedding more light on exactly how the ECM and cell interact with each other PCM-mediated control mechanisms would open new perspectives for a better understanding of healthy as well as pathological differentiation processes of chondrocytes and may also lead to the development of new therapeutic approaches.

## 5. Perspective

Regulation of cell shape and signaling from cell-cell and cell-ECM interactions are vital to the maturation of chondrocytes. Determining signaling pathways and targets downstream of these events will aid in the development of novel strategies for cartilage replacement and new approaches for regenerating cartilage and preventing and treating cartilage disorders. Still, illustrating the mechanisms integrating signals from adhesion receptors with those from growth factor and hormone receptors will contribute to a better understanding of physiologic and pathologic endochondral ossification. Complex approaches to gene mutagenesis in mice combined with advanced genomic, proteomic, and imaging tools will provide a powerful stage for rapid progress in these areas.

## Abbreviations

BFGF: Basic fibroblast growth factor
BMP: Bone morphogenic proteins
COLII: Type II collagen
COMP: Cartilage oligomeric matrix protein
CS: Chondroitin sulfate
ECM: Extracellular matrix
GAG: Glycosaminoglycan
HA: Hyaluronic acid
IGF: Insulin-like growth factor
ILs: Interleukins
ITGB1: Integrin 1
MMPs: Matrix metalloproteinases
Na^+^: Sodium ions
NO: Nitric oxide
PCM: Pericellular matrix
PGs: Proteoglycans
PKC: Protein kinase C
RA: Retinoic acid
TNF: Tumor necrosis factor
TGF: Transforming growth factor.
